# Doomed Youth: Antonio Cánovas, a Young Sportsman in Time of War in 1930s Spain

**DOI:** 10.3389/fspor.2021.577814

**Published:** 2021-02-15

**Authors:** Alejandro Viuda-Serrano, Iker Ibarrondo-Merino

**Affiliations:** ^1^Department of Education, Universidad de Alcalá, Alcalá de Henares, Spain; ^2^Faculty of Physical Activity and Sport Sciences, Universidad Politécnica de Madrid, Madrid, Spain

**Keywords:** youth, 1930s Spain, Spanish civil war (1936–1939), exile and diaspora, sports in wartime, working-class sportsmen

## Abstract

During the Spanish Civil War (SCW) 1936–1939, many young working-class sportsmen volunteered. They were both physically and politically active and some of them outstanding athletes. The search of these unknown men has just begun. *Doomed Youth* is a tribute to them and the first step toward a bigger attempt to better comprehend the role of sportsmen volunteers enlisted during the first months of the SCW, a fact that to date has received little scholar attention. Archival research, especially war combatants' family records as well as newspaper archives, oral memories of the protagonists left alive, and historical contextualization were defined as the appropriate methods to conduct the research. This paper is devoted to one of these young volunteers, Antonio Cánovas, recently dead in 2018 at the age of 98, whose life story in the 1930s and 1940s may be taken as the epitome of the young working-class sportsman of the cutting-edge regions of Spain in the first half of the 20th century: youngsters aware of their political and social rights whose dreams of social justice and active life were dashed by the war.

## Introduction

The Spanish Civil War (SCW), one of the most important world conflicts of the 20th century, resulted in the death or exile of a large number of renowned professional Spanish sportsmen. Although no systematic study has been carried out, some authors have made significant, albeit partial, efforts to assemble their names (Fernández Santander, 1990; Polo, 1993; García Candau, 2007). However, far greater was the number of young amateur practitioners who lost years or lives in the war and its consequences. The activities of many can be traced by carefully trawling newspapers of the period, but none of them have been the subject of in-depth historical research. Our study sheds light on one such life-story that could be taken as an archetype of thousands of working-class youngsters in 1930s Spain.

The main objective of this research was to expose the untold story of Antonio Cánovas as an example of politically engaged young popular athletes at a turning point in Spanish and European history. This concept of “popular” is a core and essential idea to understand sport as a working-class phenomenon, what authors such as Gounot (2005) call “working-class sports movement,” something more specific than “popular sport” or sport being practiced by workers, something clearly different from the upper-class or the middle-class concept of sport (Hargreaves, 1986; Sleap, 1998). In this sense, we can define Antonio Cánovas as a popular athlete before the war but as a working-class sportsman during and after it. The usual concept of sportsman probably does not fit with our protagonist and the other popular athletes who enrolled when war began. Examples of these athletes can be traced back from lists of combatants in battalions made up of sportsmen like the “Batallón Cultura y Deporte” or the “Batallón Deportivo.” They were not only footballers (Crónica, 1936; El Progreso, 1936; Hoja Oficial del Lunes, 1936) such as Eugenio Martínez and Julián Alcántara (CD Nacional team), Francisco Gómez and Francisco Trinchant (Ferroviaria team), and José Cotillo and Pablito (Tranviaria team) but also athletes like José Luque and Manuel Moreno (FCDO team) (El Liberal, 1937); boxers like Emilio Iglesias, José Martínez, and Ángel Fernández; cyclists like Pablo Jallola and Fernando Esparza; swimmers like Juan Borreguero; or football referees like Valentín Bravo and Tomás Balaguer (Díaz Roncero, 1936). All of them were widely unknown non-professional popular sportsmen, as in the case of Antonio Cánovas.

The authors will manage to cope with some other specific objectives on this paper. First, to discuss about sport as a multifactorial social construction that shapes reality in multiple and overlapping ways, but which is also influenced by that reality, specially by adverse life events as a civil war. In highly unstable settings twisted by such events, sport may be used as a tool to maintain a sense of coherence and meaning and may facilitate the socialization processes during or after radical life changes (Michelini, 2018). Second, to reflect on the utility of sport and physical condition for war and their immediate and indirect benefits for the military (Mason and Riedi, 2010).

**Figure 1:**
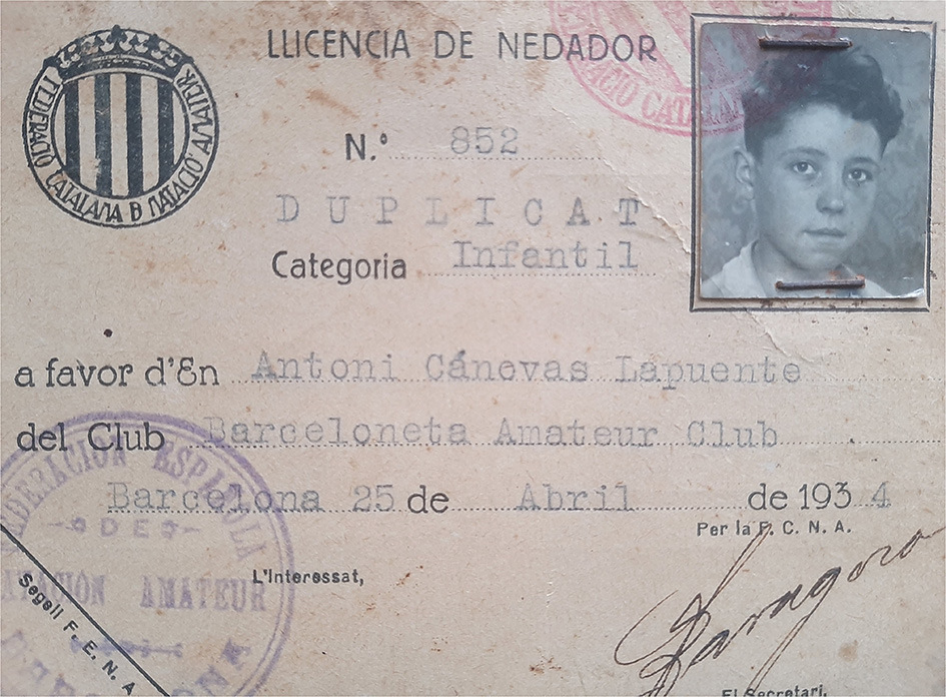
Antonio Cánovas BAC swimmer's license of children's category (1934). Source: Cánovas family archive.

**Figure 2:**
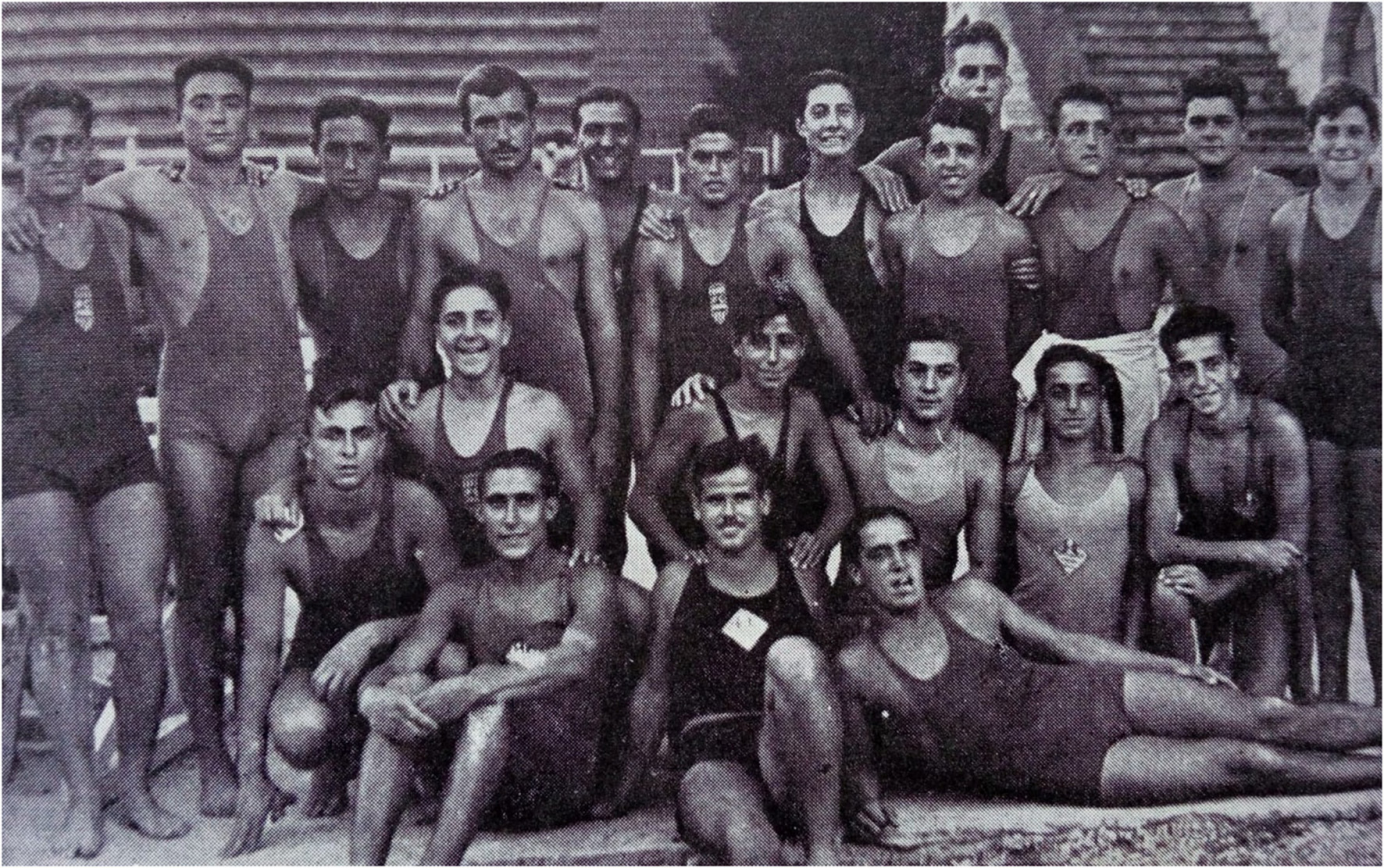
Antonio Cánovas (standing fifth from right) and his BAC teammates (1935). Source: Cánovas family archive.

**Figure 3:**
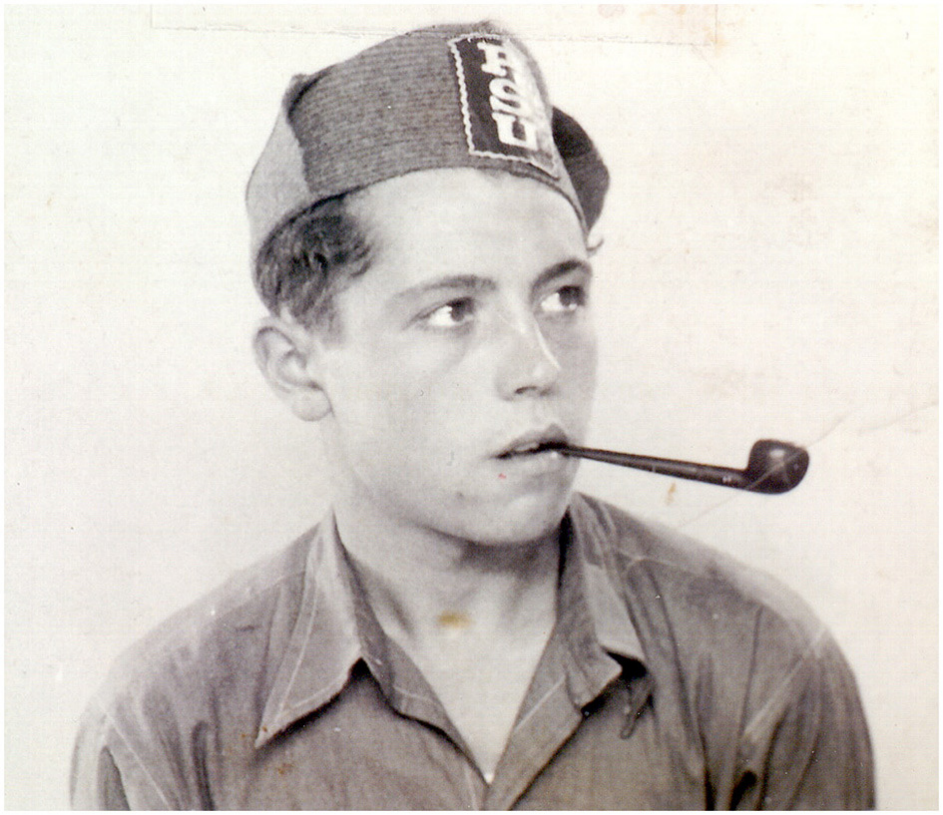
Antonio Cánovas at the beginning of the civil war (1936). Source: Cánovas family archive.

**Figure 4:**
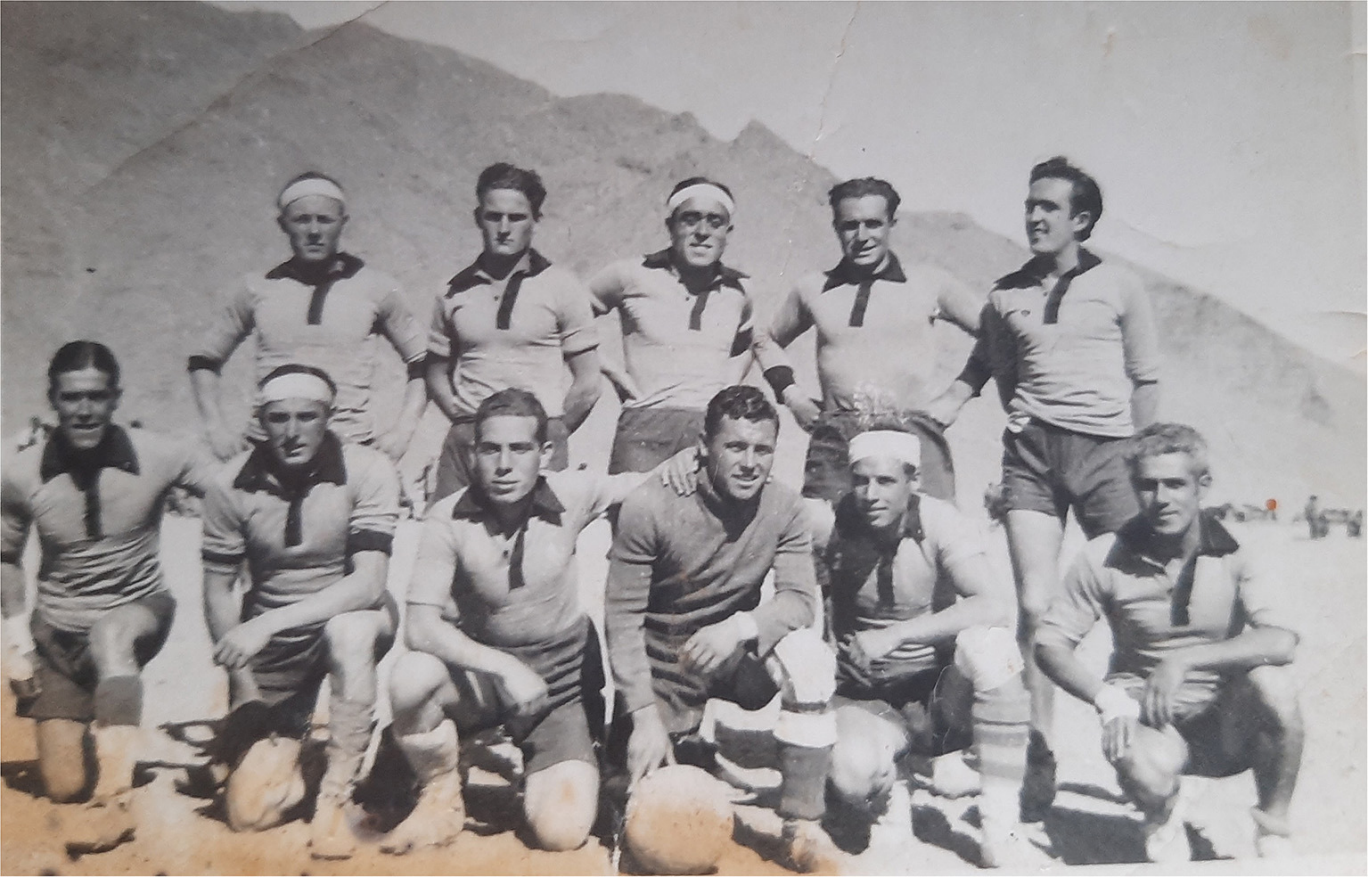
Antonio Cánovas (the crouching man with dark jersey) and his Bou Arfa concentration camp teammates (1941). Source: Cánovas family archive.

Regarding research methods used in this paper, as historical sources of any kind only provide a fragmented and partial view of the past, they should be just the point of access to that past, which must be completed with other reliable sources. This is what Day and Vamplew (2015) call the process of “complementarity, synergy and triangulation.” As these authors assume, the process of converting the past into history involves finding data, judging their validity, and accurately presenting them through a historical narrative. Being archives the main traditional source of information for historians (Johnes, 2015), authors have had the invaluable support of Antonio Cánovas' family. It was important to count not only on personal memorabilia, especially letters, but also photographs, the most used visual medium for sports historians (Huggins, 2015), identity cards, or team badges, as well as oral memories recounted by Cánovas to his relatives. We will identify these contributions into the text as part of the “Canovas family archive.” Among the dozens of letters Cánovas wrote to his brother Alfons and his family from 1936 to 1962, six were chosen to be used in this paper for their relevance about Cánovas involvement in sports. Among many photographs owned by Cánovas family, three were selected to better illustrate the Antonio's most important life stages: his first steps in sport and swimming activity during childhood (Figure 1), his first competitions (Figure 2), his involvement in the civil war as an adolescent (Figure 3), and his engagement in sport, mainly football, during his imprisonment after war (Figure 4).

Secondly, even if press narratives are not neutral and newspapers are not sources of plain fact (Hill, 2006), historians of sport, especially contemporary historians, have usually resorted to the press. Authors have traced newspapers from Spain, France, and North Africa to sustain archival sources. Translation of newspapers was carried out by the authors, native speakers of Spanish and fluent in English and French. Just a few literal quotations were eventually used. Periodicals were collected from both physical and digital Spanish and French archives:

- Biblioteca Virtual de Prensa Histórica (BVPH) of the Spanish Ministry of Culture and Sport (https://prensahistorica.mcu.es): “El liberal,” “El Progreso,” “Hoja Oficial del Lunes,” and “La Libertad.”
- Bibliotèque Numérique (GALLICA) at the Bibliotèque Nationale de France (https://gallica.bnf.fr): “L'Echo d'Oran” and “L'Humanité.”
- Center de Documentació Antiautoritari i Llibertari (CEDALL) at the Biblioteca de Catalunya (http://www.cedall.org): “Tierra y Libertad.”
- Hemeroteca Municipal de Madrid (HMM), physical archive: “Ahora. Diario de la Juventud.”
- Hemeroteca Digital (HD) at the Biblioteca Nacional de España (http://www.bne.es/es/Catalogos/HemerotecaDigital): “Crónica,” “Estampa,” “Mundo Gráfico,” and “Mi Revista.”
- Hemeroteca Digital de El Mundo Deportivo (HDMD) (https://www.mundodeportivo.com/hemeroteca): “El Mundo Deportivo.”
- Hemeroteca Digital de La Vanguardia (HDLV) (https://www.lavanguardia.com/hemeroteca): “La Vanguardia.”

Press sources will enable us to gain empirical support for the third historical source, a relatively under-used one, oral history. This method, with some concerns about possible poor memory, mis-remembrances or even nostalgia of the subjects interviewed can be invaluable if situated within a well-contextualized and well-triangulated historical narrative (Skillen and Osborne, 2015). We have used the last memories of Antonio Cánovas, overwhelmingly alert at 98 years old, interviewed by the authors in Barcelona on 12 August 2018, a few months before his death, to be checked out via a complete contextualization using dozens of research publications and some primary sources of Cánovas contemporaries, being the most important of them his brother Alfons Cánovas (2015).

Following an initial analysis of the socio-political context, especially involving sports, as well as of main concepts such as youth and war, we offer a chronologically coherent narrative (Day and Vamplew, 2015) to gain legitimate knowledge of society, moving away from simply reporting data but developing understanding of social phenomena through an adequate contextualization of biographical narratives (Oldfield, 2015). We will analyze Antonio Cánovas' life as a child before the civil war, as a soldier during the conflict, as a refugee and a prisoner after the war, as a militant communist in adulthood, but above all as a popular sportsman during his entire lifetime. Finally, we will discuss and reflect on sport as a multifactorial tool for shaping and be shaped by reality, as well as on the importance of our protagonist as the epitome of the young working-class sportsman of the cutting-edge regions of Spain in the turbulent 1930s.

## Context

### Sport in Times of War

From a historical point of view, the relevance of sport in modern warfare requires further study and attention from historians. Mason and Riedi (2010) claim that it was during World War I (WW1) that sport came to play a major role in armies as a form of social control and a means of training. Sports competitions between regiments were common, as well as betting and athletic challenges (Mason and Riedi, 2010). However, equally remarkable is the participation of sportsmen in battle. In many cases, they formed specific units, battalions of athletes. Studies have focused mainly on footballers who fought and died in WW1, especially in the British armed forces (Luedtke, 2012; McCrery, 2014a). Specific football battalions have been studied by different authors (Milner, 1991; Nannestad, 2002; Alexander, 2003; Riddoch and Kemp, 2008). Cycling and skiing have been particularly relevant to the war. In the case of the bicycle, numerous battalions of specialized cycling soldiers have been used in armies since the late 19th century (Fitzpatrick, 1998). During WW1, cycling battalions existed in practically all armies, including British (Martin, 1944), German (Stone, 2015), Canadian (Glenn, 2018), French (Allen, 1935), Belgian (Pawly and Lierneux, 2009), and Australian (Wilson, 2002) armies. Skiing is also of clear military utility. In the French army, there had been specialized battalions (*Chasseurs Alpins*) since the 19th century, just as in the Russian army. During WW1, there were units of Austrian, German, Italian, and French skiers and mountaineers, and during the Second World War (WW2), skiing was prominent in the Soviet, Norwegian, and Finnish armies. German (Williamson, 1996), Italian (Bull, 2013), and American (Shelton, 2003) armies had skier and mountaineer units. Battalions of other sports were also formed during WW1. In the British army, this was the case with rugby (Woodall, 2000; Collins, 2002; McCrery, 2014b), although there were also general sports battalions (Ward, 1920; Bilton, 2014). Australia also set up sports battalions (Phillips, 1997; Blackburn, 2016). In Canada, although there were no dedicated sports battalions, many hockey (Wilson, 2005) and baseball players (Horral, 2001) enlisted. In the Irish army, there was a battalion made up of rugby players (O'Callaghan, 2016).

Sports battalions in Spain have a shorter history. It did not participate in WW1, but from the early 20th century, thought was already being given to the importance of physical exercise in the army and its military use. Thus, in the first few decades, manuals for military cycling, military gymnastics, and physical education appeared. Before the civil war, cycling units had already been introduced in the Spanish army, such as the *Batallón Ciclista* created in 1931 (Huerta, 2016). During the war, thanks to the development of associative and workers' sports during the 1920s and 1930s, sports battalions held some prominence on the Republican side. These included the following: the *Batallón Deportivo* (García Candau, 2007), the *Batallón Ciclista Enrique Malatesta* (Ferragut, 1937), and the *Batallón Alpino del Guadarrama* (Arévalo, 2006) in Madrid; the *Batallón Ciclista* in Valencia (De Luis, 2019); the Alpine Militias in Barcelona (Flores et al., 2008); or the 96th Mixed Brigade, called the *Brigada de los Toreros* (Bullfighters' Brigade) in Murcia (Pérez, 2005). The war efforts of the Federación Cultural Deportiva Obrera (FCDO), the Spanish workers' sports organization, which created the *Compañía FCDO del Batallón Joven Guardia* (Comité Nacional de la FCDO, 1936a) and the *Batallón Cultura y Deporte* (Ibarrondo-Merino, 2019), were also noteworthy. There were many other battalions during the SCW, especially those made up from enlisted young volunteers.

### The Political and Sports Background

Political dynamism characterizes the 1920s and 1930s Spain. In about a decade, its inhabitants would see a major shift from a “dictatorship with king” (Juliá, 1999) headed by General Primo de Rivera (1923–1930) and being the king Alfonso XIII (the great-grandfather of the current king, Felipe VI) to an unstable republic (1931–1939), to finally dash against a ferocious and long-standing dictatorship (1939–1975) imposed by reactionary forces (Tuñón de Lara, 1981) after a long civil war prelude to the WW2. The advent of the Republican democratic regime came through municipal elections that would peacefully relieve Alfonso XIII (Tuñón de Lara, 1981; Brenan, 2011; García de Cortázar and González, 2016), giving way to a progressive legislation that would affect both agricultural, labor, and territorial sphere and receive broad support even from sectors such as the clergy or the army (Tamames, 1973; Vilar, 1978). Although this Republican experience has gone down in history linked to progressive ideas, it also had a conservative period (1934–1936) in which conservative measures were noted, an issue that triggered a popular revolt in Asturias and a subsequent repression (Tuñón de Lara, 1981; Brenan, 2011). However, this stage steeped in repressive episodes and government corruption would eventually regrant the government to progressive forces that unleashed the outbreak of civil war following a right-wing coup d'état (Jackson, 2005).

Internationally, the main players in the field of sport during the first third of the 20th century were international workers' sport and the Olympic movement (Pujadas and Santacana, 1990). In 1936, the strategic turn taken by the Communist International (Gounot, 2005) led to the union of the workers' sports sectors—thitherto divided between the more socialist ones aligned around the International Workers' Sports Union and the more communist ones aligned around the Red Sports International (RSI)—in order to join forces and take on the official Olympic movement. The most relevant event in the calendar of the workers' sport was the proletarian alternative to the Olympic Games, the Workers' Olympiads, held regularly during the 1920s and 1930s in Europe (Jones, 1988). It is in this context that the People's Olympics (PO) of Barcelona (Stout, 2020), one of the most significant and influential events in workers' and popular sport at international level, was held, in response to the choice of Berlin as the venue for the 1936 Olympic Games, to the detriment of, precisely, Barcelona's candidacy. The IOC's inaction in the face of Hitler's arrival to power in 1933 and the decision not to change the venue sparked an international movement initiated in the USA by the Amateur Athletic Union (AAU) that would lead to the creation of the Committee on Fair Play in Sports and to the idea being aired of boycotting Berlin. In Europe, the protest movement was orchestrated from the Anti-Fascist Conference in Paris, which agreed in late 1935 on the constitution of the International Committee for the Defense of Olympic Ideals (Pujadas and Santacana, 1990; Gounot, 2005). Barcelona had not only been a candidate to organize the Olympic Games on several occasions (Mercader, 1987) but also boasted a series of political and social characteristics and sporting infrastructures that made it the perfect host of such an event (Colomé and Sureda, 1995). Although these anti-Berlin sentiments coincided with the intentions of the RSI and were influenced by it, the PO was an event that cut across the currents opposed to fascism and was located in a different place not only from the official Games but also from the Workers' Olympics and the so-called Spartakiads (Pujadas and Santacana, 1990; Colomé and Sureda, 1995; Gounot, 2005).

Sport in Spain had begun to thrive in the 1920s in a modernizing process, albeit late in Europe, whose foundations rested on a nascent sports mercantilism in sports such as football, boxing, and cycling and the growing development of the sports press (Bahamonde, 2011). This process was particularly important in the cities, especially in the Basque Country and Catalonia, in what Pujadas (2011) calls the “sports explosion” and resulted in an extension of sports associationism to the most popular sectors of society, the working classes, rural areas, and women. In fact, an alternative discourse emerged that created a transversal popular sports movement with roots in athenaeums, gymnasiums, sports clubs, trade unions, or political party youth movements. In all these developments, there were two reference organizations: the FCDO, on the national level (Ibarrondo-Merino, 2019), and the Comitè Català pro Esport Popular (CCEP) in Catalonia (Colomé and Sureda, 1995; Gounot, 2005).

FCDO deserves further consideration for its key role on the sports context being probably the most relevant organization in Spanish working-class sports. It emanated from the previous FDO (Federación Deportiva Obrera) founded in Madrid in 1931 (De Luis, 2019). That organization came to fulfill expectations of socialist sectors of sport showed an unofficial, working-class, and progressive interpretation of the sport phenomenon as a utilitarian activity to gain political awareness, opposed to the official, capitalist idea of sport as a lucrative spectacle or a simple pastime. Both visions turned into almost antagonistic ideas of sport in Spain. Internal disputes ended up leading to the formation of the Federación Cultural Deportiva de Castilla La Nueva in 1932 (Pujadas, 2011) including the city of Madrid. This organization would merge back with the FDO by 1933 during the term in FDO office of Manuel Vento (De Luis, 2019). This new Federation would be the FCDO that channeled the most part of the working-class sport during the 1930s and extended throughout Castille as well as the Andalusian, Galician, Asturian, Basque, or Catalan territories, with little implementation in Catalonia (Pujadas, 2011) where the Catalan branch of FCDO became even autonomous (Gounot, 2005). The FCDO, which developed sports sections in different disciplines like football, athletics, or swimming among others (De Luis, 2019), stood out for the hegemony of socialist and communist ideas inside the organization that determined its sports policy until the end of the civil war.

Sport in Catalonia had undergone a dramatic change since the beginning of the 20th century, with the emergence of a sporting concept that was not only different from the rest of Spain but also different from the rest of Europe; it was forward-looking and influenced by progressive and also Catalanist ideologies. A discourse developed that was linked to citizen and class awareness, as well as to the recovery of Olympic values such as fraternity, solidarity among young people, mutual respect, and equality. Various newspapers such as *La Nau* (1927), *La Nau del Sports* (1929–30), or *La Rambla* (1930) were key propagators of this ideology, according to which doing sport was doing politics and being patriotic. It was an ideology nourished by a citizenry aware of its rights and far removed from the elitist conception of aristocratic sport, and its generalization came with the Second Republic, which created the right conditions in a favorable terrain such as Catalonia (Pujadas and Santacana, 1990). In these circumstances, the youth sectors of the Catalan popular classes had the opportunity to enjoy their free time more and to become members of various gymnastic and sports societies. The CCEP was the political base that supported this Catalan sports movement, which carried on the previous activities of several associations such as the *Ateneu Enciclopédic Popular*, the sporting section of the *Center Autonomiste de Dependents del Comerc*, the *Club Femení* *i de Esports*, or the *Center Gimnástic Barcelonés*. The CCEP brought together most of the sports societies in Catalonia, although it was only short lived due to the civil war, which changed the priorities of the sportsmen and women, many of whom were politically engaged and participated actively in the war. That is the case of Antonio Cánovas, the main character in this paper.

During the SCW, sport was used mainly for propaganda purposes (López, 2019). On the Republican side, between 1936 and 1938, sports festivals were held to promote and support the Republican cause, war victims, blood hospitals, and anti-fascist militias (Domínguez and Pujadas, 2011). In Catalonia alone, Pujadas (2007) identified more than 60 such festivals until the fall of Barcelona in January 1939. Although most were dedicated to football (39), there were also cycling (10), swimming (3), athletics (3), or boxing competitions (2), among others. Likewise, Spanish sportsmen, most of them Catalans, attended various sporting events organized by the workers' movement during the war, especially the Antwerp Workers' Olympics (García Candau, 2007; Arrechea, 2019) and the events organized by the *Fédération Sportive et Gymnique du Travail* (FSGT) in France (L'Humanité, 1937). Pro-Franco rebels, however, from the beginning of the war used sport as a diplomatic weapon (Domínguez and Pujadas, 2011), a usual Francoist strategy during the next years (Viuda-Serrano, 2010), with the clear intention of legitimizing the rebel institutions at an international level, creating a double sporting officialdom with governing bodies different from the Republican ones. The rebels were victorious in this war too, for in 1937 they achieved international recognition for the new Spanish Football Federation from FIFA and for the new Spanish Olympic Committee from the IOC.

### Youth, Politics, War, and Sport

Talking about youth as a whole is complex, since it is a very large and heterogeneous group of people with varying ages, as well as different demographic, economic, and cultural contexts, which depend on different national and local conditions. Nevertheless, in Europe, we can speak of the emergence of two different youth traditions throughout the 19th century, that of the working class and that of the middle class (Gillis, 1981). These came into conflict during the first half of the 20th century, when there was an unprecedented political mobilization of young people (Getman-Eraso, 2011). In the case of Spain, belated industrial and urban development, the key influence of the Catholic Church, and the exceptional nature of the more industrialized Basque Country and Catalonia as pioneering regions (Merino et al., 2018) conditioned the entry of young Spaniards into public life.

The ideas of modernization ushered in by the 20th century coalesced in the Spanish Republican project of the 1930s, which was supported by both the working and the middle classes and opposed by the upper class and the Catholic Church, which had enjoyed economic and cultural hegemony until then. The outbreak of the civil war in 1936 made clear the determination of the privileged classes in Spain to resist the process of social, political, and cultural modernization undertaken by the Republic (Boyd, 1997). Therefore, the clash of traditions in Spain was not, as in the rest of Europe, between young people from the middle class and the working class but between, on the one hand, young Catholics, educated in conservative values in a process undertaken with brio at the end of the 19th century and, on the other hand, young lay people, immersed in movements of educational renewal aimed at combating illiteracy, extending compulsory education, and promoting new educational methods (Merino et al., 2018).

The growing radicalization of left and right—there is knowledge of over 60 political youth organizations during the Second Republic (Casterás, 1974), especially from 1934 onwards—contributed to the increase in social tension during the years preceding the civil war (Getman-Eraso, 2011). University student movements, too, were divided on the political spectrum and often clashed with each other and with the authorities (González Calleja, 2005). Spanish leftist youth organizations were able to join against a common enemy, fascism, facilitating in April 1936 the merger of socialist and communist youth as the *Juventudes Socialistas Unificadas* (JSU), which did not happen between the same adult parties (Souto, 2004). Even more, young people are bellwethers of social change and reflect, in rather dramatic fashion, the struggles taking place in the larger society (Braungart, 1984) and usually arise in periods of crisis or radical change.

Being mobilized by the youth organizations, youngsters played a significant role in the Republican war effort (Souto, 2013). Of the three million people, with an average age of 28, who fought in the civil war, 7–8% were volunteers (Matthews, 2012). Most of them were very young and quite a number were under 18, despite the fact that the legal age of majority in Spain was 21 at the time. It is well known that the case of Antonio Cánovas was not uncommon, and many underage boys were encouraged to join. From the very beginning, premilitary physical education was established for adolescents aged 15–18 who were not permitted to enroll in the army (Consejo Nacional de Educación Premilitar Física y Cultural de la Juventud, 1937). Even boys born in 1921, the so-called “quinta del biberón” (baby-bottle generation) because of their youth (Molero, 1999), were officially called up owing to the need for soldiers on both sides of the conflict.

Connections between youth and sport were also clear during the conflict and was usually highlighted in the youth periodicals and publications that proliferated during the first months of the war. Physical activity acquired a distinct utilitarian and military nature linked with the previous political awareness that characterized sport in the 1920s and 1930s when doing sport was doing politics and being patriotic. According to these publications, the main objective of sports in wartime is gaining physical fitness of soldiers, being the time of militarizing sport to help in the war effort, because “who is better physically prepared (for war) than we (the sportsmen) are?” (Lacomba, 1936). The stated goal of the “sporting movement” inside the Republican Army was to create sport teams “in each Battalion, Company and Section” and set regular competitions to train soldiers (Ahora. Diario de la Juventud, 1937). To sum up, “the true sportsmen must be in the front line and this is not the time for trivial pastimes” (Comité Nacional de la FCDO, 1936b).

## Antonio Cánovas, a Young Sportsman in Time of War

### The Child Swimmer

Antonio Cánovas was born in 1920, the son of two Murcians who emigrated to Catalonia with their families when they were 17 and met in Barcelona. He had four brothers, two younger than him, one 2 years older than him, Alfons, and another 7 years older, Juan. He always had a special relationship with Alfons, which himself describes in his book of memories (Cánovas, 2015). He went to the municipal school in the Barceloneta district. When he was 11 years old, he was a direct witness on 14 April 1931 of the proclamation of the Second Republic in Barcelona. In 1932, due to lack of work, his parents moved to Guadalupe, in Murcia, where he was born, taking him and all his brothers with them (Cánovas, 2015). His father bought some land with the intention of cultivating it. Antonio was in charge of collecting fertilizer for the crops with an old donkey. The Murcia experience was short-lived and the whole family returned to Barcelona in 1934. He was 14 years old.

Through his brother Alfons, who was a member before him, Antonio joined the *Barceloneta Amateur Club* (BAC), a workers' swimming club founded in 1929 in a working-class neighborhood. His teammates called him “El Puça” (Flea). He competed in BAC's first international match against the “Star Olympique” (Marseilles) on 24 September 1935, on a trip by the French club to Catalonia, where they competed in swimming and water polo with several Catalan teams (El Mundo Deportivo, 1935; La Vanguardia, 1935). After the civil war, the club's name was changed to *Natació Barceloneta*. The fact that he would never forget that club can be seen in the many letters he wrote from exile to his brother Alfons (Cánovas family archive, 2020), who became the president of the club years later in 1952 (Cánovas, 2015).

In July 1936, Antonio participated in the Catalan swimming championships in the children's and beginners' category, where he swam the 200- and 400-m breaststroke in the Montjuïc pool, the only one in Barcelona which was 50 m long (Cánovas, 2018). The competitions ended at midnight on 18 July. The next day, he was due to return for the PO opening ceremony. The BAC, which was associated to the CCEP, was one of the clubs that had worked hardest on the organization of the international event. Antonio was to have formed part of the BAC team that would compete at the PO, after passing the club's selection tests (he was a substitute in the breaststroke tests), and he took part in the rehearsals for the opening ceremony planned for the 19th, which was eagerly awaited by many Barcelona residents (Stout, 2020).

On his way home that night, he saw strange troop maneuvers in the Captaincy General's office and heard some shots. His father said to Antonio and his brother, “Do what you want, I can't stop you. But whatever you do, do it for the people, never against them” (Cánovas family archive, 2020). He was a member of the anarcho-syndicalist *Confederación Nacional de Trabajadores* (CNT), the leading workers' union in Catalonia with 137,000 members in 1936 (Calero, 2009). After the military coup, the CNT was transformed from a trade union organization into the governing body of a large part of the rear guard economy and, after entering government, into a political body representing the workers in the Republican institutions (Vadillo, 2019).

In the early hours of the morning, they heard shots and gunfire in the street. In fact, the orders of the military uprising were to leave the barracks and take to the streets at 4 a.m. to take control of the important centers of power in the Catalan capital (Martínez Bande, 2007). Antonio took to the streets with his brother Alfons. There were soldiers and barricades blocking the way. It was already dawn when they arrived at the Captaincy General's office where they received one rifle for the two of them. They took part in positioning a cannon that fired directly at the facade of the military government and achieved the surrender of the rebels in Barcelona (Cánovas family archive, 2020). One of the Cánovas brothers' friends, who was also a swimmer in the BAC, died there. The CNT militants and many Barcelonans fought in the streets with the few weapons they had, and their actions were decisive in frustrating the military coup in Barcelona (Vadillo, 2019).

The PO did not take place. Most of the athletes and journalists who had arrived in Barcelona for the event were evacuated by their countries in the following days, although some of them remained in Spain to join the International Brigades that fought for the Republic. Each political party created its own militia groups and many young people close to socialism, communism, or anarchism (Getman-Eraso, 2011) led the large number of volunteers who fought from the very first moment against the coup d'état (Tierra y Libertad, 1936). On 20 July, Antonio, like many of his friends in the BAC, was the first of his brothers to volunteer in the militias of the *Partit Socialista Unificat de Catalunya* (PSUC), a party of Trotskyist and Catalanist ideology founded at the beginning of the civil war (Puigsech, 2002), another member of which was George Orwell.

The youngster, who until that moment had remained on the sidelines of the political conflict and focused on swimming, the same boy who very recently played on a donkey while helping his father on a vegetable patch in Murcia, suddenly found himself caught up in military training at the Carlos Marx barracks (Rubio, 1936), where the PSUC's military recruitment and training office was located (Closa, 2011). During the following years, the aptitudes of a promising swimmer and the hopes of a young sportsman were irreversibly shattered by the reality of war.

### The Boy Soldier

During the civil war, Antonio saw action on four different fronts (Mallorca, Aragón, Madrid, and Teruel) and participated in three of the most important and bloodiest battles of the entire conflict (Belchite, Teruel, and Ebro). After just over a week of military training, he joined the Carlos Marx Column (Alpert, 2013). Recently created in July 1936 in Barcelona, it had some 2,000 soldiers, members of the *Unión General de Trabajadores* (UGT), a Marxist trade union, and the PSUC (Gabriel, 2011). Most of the Carlos Marx militiamen were young sportsmen, “because young people are more inclined to go forward” (Cánovas, 2018). On 1 August 1936, they were sent to the Mallorca front with a contingent of some 8,000 militiamen, most of them communists, under the command of Captain Alberto Bayo. In the early morning of August 16, they disembarked in Mallorca (Bayo, 1987). In spite of outnumbering the rebels, the inexperience of the young soldiers and a series of poorly planned actions ended in the operation's failure (Aguilera, 2015). During these early days of the war, Antonio experienced the worst moments of his tragic experience. One August night, in the trenches, one of his young comrades died in his arms, calling out desperately to his mother (Cánovas, 2018). After little more than 2 weeks, the column withdrew from the island of Mallorca on September 4, embarking 3,000 soldiers on a hospital ship bound for Barcelona and 4,000 on a cargo ship bound for Valencia (Martínez Bande, 1989). However, in the haste of the escape, they left behind some soldiers and equipment. Antonio had to swim out while being shot at with machine guns from the beach. He was picked up by the Republican naval vessel Jaime I and ended up disembarking in Valencia, from where he returned to Barcelona (Cánovas, 2018). His swimming skills had saved his life in his first taste of war.

After Mallorca, the Carlos Marx Column regrouped in Barcelona and was sent to help in the defense of Madrid on 27 September 1936 (Berger, 2017). He fought in the Casa de Campo, where he served as a liaison carrying communications between trenches for the different companies on the front. At the end of 1936, he returned to Barcelona from where he was sent to the Aragon front. From May 1937, following the reorganization of the Republican army (Salas, 1973), the Carlos Marx Column became the 27th Division, known as “La Bruja” (The Witch) for its mobility and efficiency during the war (Soriano, 1989; Closa, 2011), a shock force with no fixed location, which went where it was necessary to reinforce the trenches. It included the 122nd, 123rd, and 124th Mixed Brigades (De Aragón, 1938; Maldonado, 2007). Antonio was assigned to the 122nd. He participated in the battle of Belchite, from 24 August to 6 September 1937 and was then sent to the Teruel front, where he fought in the offensive on the city from 15 December 1937. The Republicans lost the battle in February 1938. On January 19, in Visiedo, he met up again with his two brothers, Juan and Alfons. That very same day, his father was killed in an Italian bombing raid on the port of Barcelona while working in his small vegetable patch (Cánovas, 2018).

After Teruel, Antonio's brigade, which had suffered many casualties, was deployed in the rear, although shortly afterwards he was sent to the Battle of Ebro, the last major battle of the civil war. Antonio was promoted to sergeant at the age of 18. He was a true veteran despite his youth. He took part in the Republican offensive on the Ebro on 25th July 1938. After an early string of victories, the battle of Ebro became one of the attritions that the Republicans eventually lost on 15 November 1938 due to insufficient supplies and transport. The defeat at the Battle of Ebro meant that the Republican army had lost the war for good. Antonio neither returned to Barcelona out of precaution nor was he able to see his family (Cánovas family archive, 2020). His aim was to flee to France with the barely 40 soldiers from his brigade, of about 3,000, who were still alive. They were constantly bombarded and attacked on their journey through the villages of Catalonia. They advanced through rural areas and reached the French border, which they crossed at Le Perthus, on February 12, 1939 (Cánovas, 2018).

### The Young Prisoner

More than 500,000 people lost their lives in the SCW, whether in action at the front or victims of repression in the rear guard (Thomas, 2001). However, the figures for exile are even higher; 700,000 Spaniards fled during the war, of whom almost half a million fled to France between late January and early February 1939 (Rubio, 1997). Faced with pressure from hundreds of thousands of refugees, and after the failure of negotiations with Franco to create a neutral space on Spanish soil under international control, France opened the border on January 27, although only for women, children, the elderly, and the sick. Men of military age had to wait until 5 February (Alted, 2005). They were forced to surrender their weapons to the French authorities and had all kinds of vehicles and belongings seized.

In February, several concentration camps were hastily set up on the beaches near the border: the sand was fenced in and soldiers were posted to watch over them. Antonio was first held at the camp of Argelès-sur-Mer (Pyrénées-Orientales). For the first few days, there was nothing to eat and tens of thousands of Spaniards and some members of the International Brigades were literally left lying on the beach in cramped conditions (Coale, 2014). At the beginning of March 1939, a camp was opened in Agde (Hérault), where Antonio was sent, along with 24,000 other exiles, most of them Catalans (Parello, 2011). Not only did the appalling living conditions in the concentration camps drive many to return to Franco's Spain but they were also actively pressured into repatriation through Francoist and French propaganda campaigns inside the camps (Coale, 2014). Some 50,000 exiles would eventually cross back into Spain during the month of February. However, at the age of 19, Antonio knew that if he returned, at best he would be forced to do several years of military service in Franco's army; at worst, he would be tried for sedition and, having been a volunteer and holding the rank of sergeant, might be sentenced to death or summarily executed (Cánovas, 2018).

He was better off at Agde. He took part in sporting activities such as athletics and met several prestigious Catalan athletes who had been selected to take part in the PO: Jaume Àngel, Spanish 1,500-m champion in 1930 and 1935, Catalan 1,500-m record holder and working class athlete who took part in the 1937 Workers' Olympics (Arrechea, 2019) and other workers' competitions (L'Humanité, 1937); S. Puig, Catalan 100-m champion; Francisco Lorenzo, high jumper and pole vaulter from Barcelona; the Tugas brothers, José and Felipe, throwers and runners from Badalona; and J. Valls, 100-m sprinter (Etayo, 1992). He remained at Agde until the official closure of the facility in September 1939 (Parello, 2011) on France's entry into WW2, when he was sent to the Saint Cyprien camp (Pyrénées-Orientales), where they were looking for men to work and take the place of the Frenchmen dispatched to the front. However, at the end of 1939, as a volunteer worker for a company under French military command, he was sent to the naval base in Brest to build an arsenal in the port. He remained there until the Germans reached the city gates on June 19, 1940, when troops, the government, and any civilians who were able left the city without a fight (Cointet, 2017). In his escape bid, Antonio posed as a French sailor and stowed away on one of the ships leaving the port. Although the German bombardment did not succeed in sinking his ship like others, he was discovered by the French sailors and shackled in the hold (Cánovas, 2018). After several days at sea, on June 24th Antonio disembarked handcuffed in Casablanca (Morocco)—not America, which he thought was the vessel's destination—and was imprisoned.

After the civil war, 10,000–12,000 Spaniards had gone into exile in North Africa, in Algeria, Tunisia, and Morocco (Vilar, 2008). Following the armistice of June 1940, under the Vichy administration, the concentration camps in North Africa had become an openly repressive military system, which turned the Spaniards in the *Compagnies de Travailleurs Étrangers* (CTE) into virtual slaves (Charaudeau, 1992). Cánovas was tried for war crimes and sentenced to hard labor in the concentration camp of Bou Arfa, a village located on the border between Morocco and Algeria, in the middle of the desert (Martínez Leal, 2016). Despite the absence of chains, fences, and bars, there could be no bigger cell than the desert itself, which surrounded the camp on all sides. There he worked on the construction of the trans-Saharan train (Gaida, 2014), the French colonial dream for northern Africa that would link Niger to the Mediterranean Sea. The authorities saw a great opportunity for finding manpower among prisoners and expatriates fleeing the war. Some 1,200 prisoners worked on this stretch (from Bou Arfa, Morocco to Kenadsa, Algeria), most of them Spanish exiles from Oran (Algeria). Living conditions were very hard (Gaida, 2014; Cánovas, 2018). Work began at dawn and, after a break due to the high temperatures in the middle of the day, resumed in the afternoon until sunset. They ate and slept in dirty, dilapidated tents from the colonial era, in which 8–12 people were crammed together, twice their capacity, enduring temperatures that dropped to zero degrees at night.

Although all political activity was banned, an underground communist network was created with the aim of sabotaging the work of the trans-Saharan railway. Conditions worsened when punishment companies were set up to deal with acts of sabotage by the prisoners. Those punished did the hardest work, among them Antonio because of his relations with the *Partido Comunista de España* (PCE). He was part of a cell of four people who held clandestine meetings. Among other activities, they buried shovels, rakes, or drills and overturned carts (Cánovas, 2018). Thanks to the contacts he had made, he was appointed head of the JSU, and from then on, he left the hard work of building the tracks and moved to more comfortable jobs in the village of Bou Arfa. He had a certain freedom of movement within the village, although always under close military surveillance and forced to attend daily roll calls.

They worked from Monday to Saturday and spent Sundays playing sports. Antonio remembered those Sundays as the only good thing about this time. He missed being able to swim. That is what he wrote in the letters he could send to his family, especially to his brother Alfons, who would send him sports newspapers in return and tell him about the news at the BAC. He was only 20 years old, but he felt that it had been decades since he had swum in Barcelona. In spite of everything, like many exiles, he hoped to return to Spain soon (Cánovas, 2018). In one of his letters, he addressed his former swimming partners, encouraging them with the club's motto: “*Bacallà, sec, sec, bacallà, sec, sec ¡Ib bac! ¡Ib bac! ¡Ib BAC!*” (“Cod, dried, dried, cod, dried, dried, BAC! BAC! BAC!”) (Cánovas family archive, letter from October 15, 1940). Faced with the impossibility of swimming, he began to play football. Every Sunday, he would go down to the village of Bou Arfa with other prisoners. He played as a goalkeeper and proved to be one of the best. In 1941, he was selected to play for another team from the camp and even entered the lineup for a game in Oujda (267 km north of Bou Arfa). He played other sports. He took part in an international athletics meeting in the region, becoming the 100-m and 4 × 100 relay champion and runner-up in the long jump (Cánovas family archive, 2020). The Spanish team won the championships. Whenever he could, he also produced a small sports newspaper on Sundays, which gave the results of the Spanish football league taken from the news he could hear on the radio.

Since sabotage in the prison camp was constant, in June 1942, the communist organization was discovered and reported to the French authorities, who arrested Antonio and the others involved, thus ending his 2 years in the hard-labor camp of Bou Arfa. He was taken to a police station, interrogated, beaten, and tortured. He was then transferred to Meknès, where a military tribunal accused him of sabotage at a court martial and sentenced him to 3 years in the Port Lyautey prison, known as the *maison central*, built in 1930 as the first maximum security prison in Morocco and used for prisoners with long sentences, especially political prisoners (Slyomovics, 2005). In the prison, with a capacity of 425 people, conditions were much stricter and harsher than in the desert. He was taken out to quarry stone morning and afternoon. He was kept in a cell with common prisoners, separated from the rest of the communists imprisoned with him, although there too he came across an underground communist organization. The prison routine was not interrupted until the American troops landed at Port Lyautey on November 8, 1942. Heavy Allied bombing destroyed part of the prison. Some common prisoners escaped. However, the political prisoners decided to stay in their cells. The guards shot anyone who tried to escape, and they were convinced that they would soon be released. Indeed, within a few days, the victorious American forces arrived at the prison handing out tobacco and chewing gum. They offered them freedom if they joined their army to fight on the European front, with the promise of paying them a salary and granting them American nationality (Cánovas, 2018). He refused the offer and remained in prison. He was held for eight more months, while the French collaborators were arrested, and the Americans took control of the colony. At least they no longer had to quarry but worked in an agricultural factory, a much less arduous task. In April, under pressure from French communist organizations, the work camps were closed, and in July, the inmates were released. The Spanish prisoners in Port Lyautey were also released. Toward the end of July 1943, Antonio was sent to Casablanca.

### The Militant Free Sportsman

Antonio was physically free, although he was still far from his family. The *Parti Communiste Français* (PCF), along with the *Parti Communiste du Maroc* (PCM), newly founded in 1943, organized a network coordinated through a committee for the reception of former prisoners to welcome them into the homes of like-minded families. He had to integrate himself into a society that was totally foreign to him. He found a job as a mechanic in an American army truck workshop. When not working, he attended social gatherings with other exiles. At a dance to help refugees, he met his future wife, Micaela. She was 17 and he was 23.

From this point on, he was able to return to sports. He played as a goalkeeper in the *Honneur* Division in Morocco, at first as a substitute, but later he would earn a place in the first team. In 1943, in a letter to his brother Alfons, he wrote: “Of the four games I've played, I haven't let a single goal in” (Cánovas family archive, letter from November 3, 1943). Those letters were his only contact with his family in Barcelona. Both Antonio and Alfons maintained an intense sporting life in spite of their different life paths. “Football is my destiny, I have been made some offers for next season,” Antonio wrote in another letter to Alfons in 1944 (Cánovas family archive, letter from August 1, 1944).

He remained in contact with the PCF and attended their meetings to raise money to help refugees and the clandestine struggle in Spain. He collaborated with the anti-Franco resistance in Morocco. The PCE, in which he was an activist, sent Antonio to Bordeaux to work underground, where his brother Juan already lived. Like many in exile and inside Spain, he was confident that the Franco dictatorship would fall after the allied victory in WW2. On July 1, 1946, he left Casablanca via Oran with his wife and 1-year-old son. He landed in Port-Vendres (Pyrénées-Orientales), on a false passport, posing as a PCF repatriate returning after the war. While in Bordeaux, he was required to support Spanish guerrilla activity on the French border. Under the alias of Ricardo, he infiltrated the Pyrenees rear guard. He lost all contact with his wife, Micaela, who had to return to Casablanca in September 1947 due to financial difficulties in Bordeaux where she was unable to work because she lacked official papers. Antonio's role was to act as a “passer,” escorting and transporting people and materials from France to the other side of the border. He used isolated paths to reach a specific point in Spain, leave what he was transporting, and return to France. The clandestine network was suddenly dismantled, and he had to return to Bordeaux without explanation. From there, he moved to Paris, where he was reunited with his wife and son. These were difficult times marked by hunger and cold. In that period, his work consisted in making false-bottomed suitcases to pass documents to Spain (Cánovas family archive, 2020).

In September 1948, the PCE allowed Antonio to return to Bordeaux, where his daughter Nadia was born in 1949. Micaela returned to Casablanca, but Antonio had problems with his illegal passport. To travel legally to Casablanca, he needed a job contract in Morocco that he would not get until July 1950, through a Spanish player from the *Idéal Club Marocain* football team, in exchange for playing for the club.

During the 2 years of waiting in Bordeaux, Antonio left the underground and returned to sport. He played football in Bordeaux, although it was hard to reconcile it with work. In a letter, he explained that “when you win you don't feel the blows or the tiredness until the next day you have to go to work” (Cánovas family archive, letter from February 24, 1949). In 1950, at the age of 30, he returned to swimming training to fill the void left by the enforced separation from his family. He participated in a local competition, winning the 100- and 200-m breaststroke. “The times are not bright, but it is a satisfaction after such a long time away from the pool; and winning has boosted my morale,” he said in a letter (Cánovas family archive, letter from July 3, 1950).

In Casablanca, he became a great angling enthusiast, as he wrote in a letter: “It is one of my biggest distractions right now” (Cánovas family archive, letter from August 26, 1951). He continued with athletics and football. At 31, he was in excellent shape, playing two games a week. In 1951, his team, the *Idéal Club Marocain*, was promoted to the *Honneur* Division of Moroccan football (L'Echo d'Oran, 1951). In 1952, he made it to the first team, although the following season they were relegated to *Pre-Honneur* again (L'Echo d'Oran, 1952). Antonio carried on playing sport until his return to Barcelona in 1962, after 23 years of exile.

## Conclusion

Along this paper, we have discussed sport as a pastime and leisure activity; as an effective means of getting a good physical condition; as a way to fill the void or to think of something other than extremely adverse situations, to improve life conditions, or to achieve employment; as an opportunity to travel or to meet new people; and as a way for political awareness… It is clear to us that sport not only shapes reality but is also influenced by real experiences, especially by traumatic events. Antonio Cánovas not only took advantage of his physical condition during war and exile but also used sports to deal with his imprisonment and loneliness, being sports practice a means to forge personality. This paper also states the FCDO's key role in Spanish working-class sports movement and the political boost of the Republican regime to bring Spain closer to the European sports development.

Antonio Cánovas died in 2018, at the age of 98, a few months after being interviewed by the authors. He had a long and full life although not free of obstacles. He had been swimming since his adolescence in Barcelona, the Spanish city of sport par excellence, as many Catalan laborers' and workers' children did during the modernizing process of the 1920s and especially the 1930s. He saw friends and enemies die in the SCW, like most of the nearly three million people who fought in the conflict. Furthermore, he volunteered out of political and moral convictions as all the estimated 240,000 young leftist volunteers did. He endured hunger, cold, and loneliness in the French concentration camps and was treated as a dangerous criminal, the same as the almost half a million who fled from repression between late January and early February 1939. He was subjected to forced labor in North African concentration camps, like a few thousand Spaniards, most of them Catalans, and got involved in political activism. He was imprisoned as a political prisoner in a maximum security penitentiary, and even when Allies freed North Africa, he was held for eight more months. He was an anti-fascist and anti-Franco activist who risked his life and his family's safety during the harshest post-war years out of strong convictions. He spent 23 years in exile away from his family, his friends, and his home country. But, he also practiced sports all his life, even in hard conditions, during the war, in the French and Moroccan concentrations camps, in prison, in the underground, and as a free man. He met new friends and raised a family. Neither adversity nor suffering succeeded in breaking the youthful vitality of a character forged in sport from childhood, something remarkable even in his old age.

Antonio is one of those thousands of anonymous sportsmen that neatly explain the intersection between youth, war, and sport in Spain of the turbulent and critical 1930s, whose life story may be taken as the epitome of the young working-class sportsman of the cutting-edge regions of Spain in the first half of the 20th century: a generation where youth were doomed to failure, youngsters aware of their political and social rights whose dreams of social justice and active life were dashed by the war.

## Data Availability Statement

The original contributions presented in the study are included in the article/supplementary material, further inquiries can be directed to the corresponding author/s.

## Ethics Statement

Written informed consent was obtained from the individual(s) for the publication of any potentially identifiable images or data included in this article.

## Author Contributions

All authors listed have made a substantial, direct and intellectual contribution to the work, and approved it for publication.

## Conflict of Interest

The authors declare that the research was conducted in the absence of any commercial or financial relationships that could be construed as a potential conflict of interest.
